# Supplementary material for: Application of biological and fisheries attributes to assess the vulnerability and resilience of tropical marine fish species
Source: PLoS One. 2021 Aug 17;16(8):e0255879. doi: 10.1371/journal.pone.0255879 (PMC8370639; doi:10.1371/journal.pone.0255879)
Supplement: S1 Table — (DOCX) [file pone.0255879.s001.docx]

**Supplementary Information**

MS Title: Application of biological and fisheries attributes to assess the vulnerability and resilience of tropical marine fish species

Authors: K. S. Mohamed, T.V. Sathianandan, E. Vivekanandan, S. Kuriakose, U. Ganga, S. L. Pillai and R. J. Nair

**Table S1.** List of sources used for collating life history and fishery parameters of tropical marine species of the Indian subcontinent.

| Journal/ Source | Period | Papers referred |
| --- | --- | --- |
| Indian Journal of Fisheries | 1954 -2010 | 86 |
| Asian Fisheries Science | 1988 -2007 | 2 |
| Journal Marine Biological Association of India | 1959-2010 | 32 |
| Bulletin Marine Science | 1996 | 1 |
| Indian Journal of Marine Science | 1997 -2005 | 6 |
| CMFRI Annual Reports | 1999 -2015 | 9 |
| CMFRI Bulletins | 1986 - 2010 | 9 |
| CMFRI Books | 2000-2010 | 3 |
| Proceedings of symposia | 1993 -2005 | 8 |
| PhD Theses |  | 5 |
| Journal of Indian Fisheries Association |  | 2 |
| CIFE Publication |  | 1 |
| Bangladesh Journal of Fisheries Research |  | 1 |
| Marine Fisheries Information Service of CMFRI |  | 1 |
| Asian Fisheries Science abstracts |  | 2 |
| Journal of Ecobiology |  | 1 |
| FishBase |  | Database |
| FAO Identification sheets |  | Database |
|  |  | 169 |
