# Supplementary material for: Application of biological and fisheries attributes to assess the vulnerability and resilience of tropical marine fish species
Source: PLoS One. 2021 Aug 17;16(8):e0255879. doi: 10.1371/journal.pone.0255879 (PMC8370639; doi:10.1371/journal.pone.0255879)
Supplement: S2 Table — Due to recent taxonomic revisions in some cases, the old nomenclature is also shown in brackets along with common names and the family of the species. Primary classification into 6 broad groups (teleosts, elasmobranchs, crustaceans, bivalves, cephalopods and gastropods). Further classification of the list is family-wise, alphabetically. The species numbers are used in figures (resilience and vulnerability plots). The broad habitat of the species is indicated by pelagic (P), demersal (D) and midwater (M). (DOCX) [file pone.0255879.s002.docx]

**Table S2.** List of 133 species (current nomenclature as per WoRMS database available from <http://www.marinespecies.org>) used in the IRV assessment. Due to recent taxonomic revisions in some cases, the old nomenclature is also shown in brackets along with common names and the family of the species. Primary classification into 6 broad groups (teleosts, elasmobranchs, crustaceans, bivalves, cephalopods and gastropods). Further classification of the list is family-wise, alphabetically. The species numbers are used in figures (resilience and vulnerability plots). The broad habitat of the species is indicated by pelagic (P), demersal (D) and midwater (M).

| **No** | **Current Nomenclature** | **Family name** | **Common Name** | **Habitat** |
| --- | --- | --- | --- | --- |
| **Teleosts** | | | |  |
| 1 | *Arius jella (Tachysurus jella)* | Ariidae | Blackfin sea catfish | M |
| 2 | *Nemapteryx nenga* | Ariidae | Engraved catfish | M |
| 3 | *Netuma thalassina* | Ariidae | Giant catfish | M |
| 4 | *Osteogeneiosus militaris* | Ariidae | Soldier catfish | D |
| 5 | *Plicofollis dussumieri* | Ariidae | Blacktip sea catfish | M |
| 6 | *Plicofollis tenuispinis* | Ariidae | Thinspine sea catfish | M |
| 7 | *Ariomma indicum (Ariomma indica)* | Ariommidae | Indian driftfish | M |
| 8 | *Odonus niger* | Balistidae | Red-toothed trigger fish | M |
| 9 | *Ablennes hians* | Belonidae | Flat needlefish | P |
| 10 | *Pseudorhombus arsius* | Bothidae | Large-toothed flounder | D |
| 11 | *Alepes djeddaba* | Carangidae | Shrimpscad | P |
| 12 | *Alepes kleinii (Alepes kalla)* | Carangidae | Razorbelly scad | P |
| 13 | *Atropus atropos* | Carangidae | Cleftbelly trevally | P |
| 14 | *Atule mate* | Carangidae | Yellowtail scad | P |
| 15 | *Caranx hippos (Caranx carangus)* | Carangidae | Crevalle jack | P |
| 16 | *Decapterus macrosoma* | Carangidae | Shortfin scad | M |
| 17 | *Decapterus russelli* | Carangidae | Indian scad | M |
| 18 | *Megalaspis cordyla* | Carangidae | Torpedo scad | P |
| 19 | *Parastromateus niger* | Carangidae | Black pomfret | D |
| 20 | *Scomberoides tol* | Carangidae | Needlescaled queenfish | P |
| 21 | *Selar crumenophthalmus* | Carangidae | Bigeye scad | P |
| 22 | *Selaroides leptolepis* | Carangidae | Yellow-striped scad | P |
| 23 | *Caranx ignobilis* | Carangidae | Giant trevally | M |
| 24 | *Chirocentrus dorab* | Chirocentridae | Dorab wolf herring | P |
| 25 | *Amblygaster sirm* (*Sardinella sirm)* | Clupeidae | Spotted sardinella | P |
| 26 | *Dussumieria acuta* | Clupeidae | Rainbow sardine | P |
| 27 | *Escualosa thoracata* | Clupeidae | White sardine | P |
| 28 | *Ilisha megaloptera* | Clupeidae | Bigeye ilisha | P |
| 29 | *Sardinella albella* | Clupeidae | White sardinella | P |
| 30 | *Sardinella dayi* | Clupeidae | Day's sardine | P |
| 31 | *Sardinella fimbriata* | Clupeidae | Fringe-scale sardine | P |
| 32 | *Sardinella gibbosa* | Clupeidae | Gold-striped sardinella | P |
| 33 | *Sardinella longiceps* | Clupeidae | Indian oil sardine | P |
| 34 | *Tenualosa ilisha* | Clupeidae | Hilsa shad | P |
| 35 | *Thryssa mystax* | Clupeidae | Moustached thryssa | P |
| 36 | *Cynoglossus arel* | Cynoglossidae | Largescale tonguesole | D |
| 37 | *Cynoglossus macrostomus* | Cynoglossidae | Malabar tonguesole | D |
| 38 | *Coilia dussumieri* | Engraulidae | Gold-spotted grenadier anchovy | P |
| 39 | *Encrasicholina devisi* | Engraulidae | Shorthead anchovy | M |
| 40 | *Encrasicholina punctifer* | Engraulidae | Buccaneer anchovy | M |
| 41 | *Stolephorus waitei* | Engraulidae | Spotty-face anchovy | M |
| 42 | *Harpadon nehereus* | Harpadontidae | Bombay-duck | P |
| 43 | *Istiophorus platypterus* | Istiophoridae | Indo-pacific sailfish | P |
| 44 | *Lactarius lactarius* | Lactariidae | False trevally (whitefish) | D |
| 45 | *Eubleekeria jonesi* | Leiognathidae | Jones's ponyfish | D |
| 46 | *Eubleekeria splendens* | Leiognathidae | Splendid ponyfish | D |
| 47 | *Gazza minuta* | Leiognathidae | Tooth pony fish | D |
| 48 | *Karalla dussumieri* | Leiognathidae | Dussumier's ponyfish | D |
| 49 | *Leiognathus brevirostris* | Leiognathidae | Shortnose ponyfish | D |
| 50 | *Leiognathus ruconius* | Leiognathidae | Deep pugnose ponyfish | D |
| 51 | *Photopectoralis bindus* | Leiognathidae | Orange-fin ponyfish | D |
| 52 | *Secutor insidiator* | Leiognathidae | Pugnose ponyfish | D |
| 53 | *Pristipomoides filamentosus* | Lutjanidae | Crimson jobfish | M |
| 54 | *Upeneus sulphureus* | Mullidae | Sulphur goatfish | D |
| 55 | *Upeneus taeniopterus* | Mullidae | Fin-stripe goatfish | D |
| 56 | *Upeneus vittatus* | Mullidae | Striped goatfish | D |
| 57 | *Nemipterus randalli* | Nemipteridae | Randall's threadfin bream | D |
| 58 | *Nemipterus bipunctatus* | Nemipteridae | Delagoa threadfin bream | D |
| 59 | *Nemipterus japonicus* | Nemipteridae | Japanese threadfin bream | D |
| 60 | *Grammoplites suppositus* | Platycephalidae | Spotfin flathead | D |
| 61 | *Priacanthus hamrur* | Priacanthidae | Moontail bullseye | D |
| 62 | *Rachycentron canadum* | Rachycentridae | Cobia | P |
| 63 | *Johnius borneensis* | Sciaenidae | Sharptooth hammer croaker | D |
| 64 | *Johnius carutta* | Sciaenidae | Karut croaker | D |
| 65 | *Johnius dussumieri* | Sciaenidae | Bearded croaker | D |
| 66 | *Johnius glaucus* | Sciaenidae | Pale spotfin croaker | D |
| 67 | *Johnius macrorhynus* | Sciaenidae | Big-snout croaker | D |
| 68 | *Johnius sina (Johnieops sina)* | Sciaenidae | Sin croaker | D |
| 69 | *Kathala axillaris* | Sciaenidae | Kathala croaker | D |
| 70 | *Nibea maculata* | Sciaenidae | Blotched croaker | D |
| 71 | *Otolithes cuvieri* | Sciaenidae | Lesser tigertooth croaker | D |
| 72 | *Otolithes ruber* | Sciaenidae | Tigertooth croaker | D |
| 73 | *Pennahia anea (Johnieops aneus, P. macrophthalamus)* | Sciaenidae | Greyfin croaker | D |
| 74 | *Protonibea diacanthus* | Sciaenidae | Spotted croaker | D |
| 75 | *Otolithoides biauritus* | Sciaenidae | Bronze croaker | D |
| 76 | *Auxis rochei* | Scombridae | Bullet tuna | P |
| 77 | *Auxis thazard* | Scombridae | Frigate tuna | P |
| 78 | *Euthynnus affinis* | Scombridae | Kawakawa (little tuna) | P |
| 79 | *Katsuwonus pelamis* | Scombridae | Skipjack tuna | P |
| 80 | *Rastrelliger kanagurta* | Scombridae | Indian mackerel | P |
| 81 | *Sarda orientalis* | Scombridae | Striped bonito | P |
| 82 | *Scomberomorus commerson* | Scombridae | Narrow-barred Spanish mackerel | P |
| 83 | *Scomberomorus guttatus* | Scombridae | Indo-pacific king mackerel | P |
| 84 | *Scomberomorus lineolatus* | Scombridae | Streaked seerfish | P |
| 85 | *Thunnus albacares* | Scombridae | Yellowfin tuna | P |
| 86 | *Thunnus tonggol* | Scombridae | Longtail tuna | P |
| 87 | *Epinephelus diacanthus* | Serranidae | Spinycheek grouper | D |
| 88 | *Sphyraena barracuda (S. picuda)* | Sphyraenidae | Great barracuda | P |
| 89 | *Sphyraena jello* | Sphyraenidae | Pick handle barracuda | P |
| 90 | *Sphyraena obtusata* | Sphyraenidae | Obtuse barracuda | P |
| 91 | *Pampus argenteus* | Stromateidae | Silver pomfret | D |
| 92 | *Saurida tumbil* | Synodontidae | Greater lizardfish | D |
| 93 | *Saurida undosquamis* | Synodontidae | Brushtooth lizardfish | D |
| 94 | *Eupleurogrammus muticus* | Trichuiridae | Smallhead hairtail | M |
| 95 | *Lepturacanthus savala* | Trichuiridae | Savalai hairtail | M |
| 96 | *Trichiurus lepturus* | Trichuiridae | Largehead hairtail | M |
| **Elasmobranchs** | | | |  |
| 97 | *Carcharhinus limbatus* | Carcharhinidae | Blacktip shark | D |
| 98 | *Carcharhinus sorrah* | Carcharhinidae | Spottail shark | D |
| 99 | *Glaucostegus granulatus (Rhinobatos granulatus)* | Rhinobatidae | Granulated shovel-nose ray | D |
| 100 | *Rhizoprionodon acutus* | Carcharhinidae | Milk shark | D |
| 101 | *Scoliodon laticaudus* | Carcharhinidae | Spadenose shark | D |
| 102 | *Sphyrna lewini* | Sphyrnidae | Scalloped hammerhead shark | D |
| **Crustaceans** | | | |  |
| 103 | *Acetes indicus* | Sergestidae | Paste Shrimp | M |
| 104 | *Charybdis feriatus* | Portunidae | Cross crab | D |
| 105 | *Exhippolysmata ensirostris* | Hippolytidae | Hunter Shrimp | D |
| 106 | *Metapenaeus affinis* | Penaeidae | Jinga Prawn | D |
| 107 | *Metapenaeus brevicornis* | Penaeidae | Yellow Prawn | D |
| 108 | *Metapenaeus dobsoni* | Penaeidae | Flower-Tail Prawn | D |
| 109 | *Metapenaeus monoceros* | Penaeidae | Speckled Prawn | D |
| 110 | *Nematopalaemon tenuipes* | Palaemonidae | Spider Prawn | D |
| 111 | *Oratosquilla nepa* | Squillidae | Mantis Shrimp | D |
| 112 | *Panulirus polyphagus* | Palinuridae | Mud Spiny Lobster | D |
| 113 | *Parapenaeopsis hardwickii* | Penaeidae | Spear Prawn | D |
| 114 | *Parapenaeopsis maxillipedo* | Penaeidae | Torpedo Shrimp | D |
| 115 | *Parapenaeopsis stylifera* | Penaeidae | Kiddi Prawn | D |
| 116 | *Penaeus indicus* | Penaeidae | Indian White Prawn | D |
| 117 | [*Penaeus monodon*](http://www.catalogueoflife.org/col/details/species/id/3f4b6920bc064405a96062177350908e/synonym/699a4bbbe641d9e8abe62bc4f8648446) | Penaeidae | Giant Tiger Prawn | D |
| 118 | *Penaeus semisulcatus* | Penaeidae | Green Tiger Prawn | D |
| 119 | *Portunus pelagicus* | Portunidae | Blue Swimmer Crab | M |
| 120 | *Portunus sanguinolentus* | Portunidae | Spotted Swimming Crab | M |
| 121 | *Solenocera choprai* | Solenoceridae | Mud Shrimp | D |
| 122 | *Solenocera crassicornis* | Solenoceridae | Coastal Mud Prawn | D |
| **Bivalves** | | | |  |
| 123 | *Paphia malabarica* | Veneridae | Short-neck clam | D |
| 124 | *Placuna placenta (Placenta placenta)* | Ostreoidae | Window-Pane Oyster | D |
| 125 | *Tegillarca granosa*  *(Anadara granosa)* | Arcidae | Cockle (Blood Clam) | D |
| **Cephalopods** | | | |  |
| 126 | *Sepia aculeata* | Sepiidae | Needle Cuttlefish | D |
| 127 | *Sepia elliptica* | Sepiidae | Oval-Bone Cuttlefish | D |
| 128 | *Sepia pharaonis* | Sepiidae | Pharaoh Cuttlefish | D |
| 129 | *Sepiella inermis* | Sepiidae | Spineless cuttlefish | D |
| 130 | *Uroteuthis (Photololigo) duvaucelii (Loligo duvaucelii)* | Loliginidae | Indian squid | M |
| **Gastropods** | | | |  |
| 131 | *Babylonia spirata* | Babyloniidae | Spiral babylon | D |
| 132 | *Babylonia zeylanica* | Babyloniidae | Indian babylon | D |
| 133 | *Turbinella pyrum* | Turbinellidae | Indian chank | D |
