# Supplementary material for: Application of biological and fisheries attributes to assess the vulnerability and resilience of tropical marine fish species
Source: PLoS One. 2021 Aug 17;16(8):e0255879. doi: 10.1371/journal.pone.0255879 (PMC8370639; doi:10.1371/journal.pone.0255879)
Supplement: Attachment — Submitted filename: PONE-D-20-34956_Review.pdf [file pone.0255879.s003.pdf]

2/5/2021

PONE-D-20-34956 review

Title: Application of biological and fisheries attributes to assess the vulnerability and resilience of tropical marine fish species

Overall comments:

Thank you for the opportunity to provide feedback. This manuscript was an interesting read and tackles an important issue for improving fisheries management of multiple species in Indian coastal waters. The authors demonstrate the need for such information in the introduction, and they explore the utility of using biological and fishery attributes to quantify the resilience and vulnerability to different fish species using three metrics. While I think the manuscript would be thematically appropriate for this journal, I think there are substantial issues with the level of detail and writing clarity provided in this draft, which makes it difficult to assess the authors' approach. Thus, I cannot recommend this manuscript for acceptance in PLOS ONE at this time. I provide general and specific comments below to assist in efforts to improve the manuscript.

General comments:

- In general, this manuscript would benefit from a careful proofreading, as there are numerous spelling and grammatical mistakes throughout. I highlight some of these in the *Specific comments* below. This may help the writers clarify their meaning in some sections of the paper, which will be helpful for the overall interpretability.
- There are many statements throughout the introduction and discussion that would be much stronger with a supporting citation. I think this manuscript could do a more thorough job of incorporating relevant published work.
- I do not agree that resilience and vulnerability are innately opposites, as is hypothesized here. As indicated in L652-653, you expected high R with low V (or vice versa) but did not always observe this result. This does not surprise me, since the two indices you calculate (R and V), while complimentary, describe attributes of each species/fishery that are not necessary inversely related (or related at all). At points throughout the manuscript, it is implied that R and V are inversely related, and these statements need to be adjusted to either justify this expectation by the authors or to clarify their expectations if my interpretation above is incorrect.
- A figure showing a map of the study regions (data source areas) would be helpful and could be referenced throughout the manuscript to provide better spatial context (e.g., L192-193, L870-873)

Specific comments:

L22: delete "was estimated".

L28: "large-sized" should be changed to "large".

L30-31: delete “by the species within the same family”.

L33: rather that “contradictory results”, use something like “different species vulnerabilities” or “different fish management priorities”.

L47: “species of diverse” should be “species with diverse”.

L50: delete “number of”.

L89: both mentions of “is” should be changed to “are”.

L106-107: with regard to the Hilborn et al. (2020) citation, is there any information available to allow you to describe the state of the fisheries used in this study? Are they “in poor shape” as you say?

L115: BMSY and FMSY have not been defined elsewhere in the manuscript.

L119-127: This paragraph brings up an important point. You describe 133 species, but use data from 644 stocks. Are there multiple stocks for some of these species? If so, how were data from different stocks combined for a single species?

L141-143: although the original paper is cited, I believe it would be helpful to briefly describe the attributes from the siFISH analysis that were important for the sustainability classifications you list.

L145-147: 4 values of K are listed for 3 classes.

L152: L_∞ symbology is incorrect here and throughout the manuscript (file error?)

L152: Should mention the general species/stocks that Musick (1999) uses so we can assess how comparable these approaches are.

L155: how were these thresholds identified?

L158-168: while this citation seems appropriate for this paper, there are several terms that could use some brief additional detail to help the reader follow. For example, the meaning of “adaptive capacity” is unclear. What types of variables fit this criteria?

L192-193: were these ecoregions sampled equally? It would be very beneficial to include a figure with a map showing the ecoregions from which data were collected.

L194-196: the database is not currently accessible via Google Drive.

L197: Table 1 does not seem necessary to include in the main body, especially since the INMARLH index in referenced sparingly in the manuscript. This table could easily be move to supplemental files. The last two rows in the table are included despite no papers referred and can be deleted. Is there a reason to describe this index in such detail given how little it is used in the manuscript?

L202: this point is worth bringing up in the discussion; does data availability here limit or bias the results in any way? What other attributes, if any, do you think could improve these calculations? You could go into greater detail on L561, for example.

L205-212: Table 2 should be in the supplemental file rather than the main body text.

L206, 213-216: Figures 1a & 1b do not seem necessary to me as presented. It would be good to indicate in the text, while describing the variables, the sample size (N) for each. You could likewise describe the distributions of scores (means, SDs, ranges, etc.) for each attribute in a table in the results. If you want to include such figures in the supplemental materials, the quality needs to be improved.

L224: This caption should do more to clarify what high scores for resilience and vulnerability indicate.

Table 3: The logic column would benefit from citations for many of the statements (these could also be included in the attribute descriptions that follow). Is the scoring system for landing price backwards (see also L309-310)?

L242: how many species required use of this proxy?

L267-268: clarify what is meant by “maximally distributed”.

L278-283: it is unclear how we should interpret these scores. How did you decide on cutoffs to adjust distributions to rankings?

L315-316: can delete this sentence.

L326: parenthetically indicate whether x-axis refers to resilience or vulnerability.

L343: “quickly” should be “accurately”.

L369-371: there needs to be more elaboration on your methodology. It is hard to follow what was done here.

L381: doesn't your IRV only represent Indian stocks? How comparable are IUCN data from other regions in these cases? That needs to be considered when comparing these indices.

Table 4: I like the idea of this table. To make it easier to follow, you might consider using vertical lines to separate the resilient, vulnerable, and risky sections.

L427-429: Figure 3 is very helpful for describing overall patterns. The separate plots (Figures 4-7) work better for interpretation; I suggest keeping Fig 3 and combining Figs 4-7 into Figs 4a-d. How are Figs 4a and 4b are currently split? Is there a reason for this? Make sure to reference Table 2 (or if it move to supplemental table) in the figure caption so readers can identify the species shown on each plot.

L434 and L453: You start to describe reasons for the classification…this should go in the discussion.

L471: should we be concerned that the most resilient species has an IRV of 0.52, and not closer to the theoretical maximum value? What aspects of the biological and fishery attributes for this species give it the highest IRV? Should we be concerned about Indian fish stocks overall? – all things to be considered in the discussion.

Figure 8: This figure could either be broken into descriptive statistics (for example, can include with L473-475) or would be improved by color coding the bars in a stacked histogram to show the distribution of IRV scores by family.

L481: "scores" = IRV scores?

L483-484: why use the 6 most sensitive attributes and not 1, 3, 5, etc? Justify the cutoff and selection process used.

L485-486: why evaluate only the 10 most resilient and vulnerable species, rather than compare IRV and sIRV for all 133 species? In Fig 9, the classification of species doesn't change for any of the species shown, but what about intermediate ones?

L500: why were these 11 species chosen for comparison?

L504-505: do differences in these indices show any trends across particular groups (elasmobranchs, etc)?

L540-541: this idea needs to be elaborated on further in the discussion.

L576: curious if there is any concern about gear bias in the results. Related to this, is gear regulation (e.g., changing mesh sizes) an option for targeted management of at-risk/vulnerable/less resilient fisheries?

L586-587: were there any temporal trends in the other variables besides Er?

L609-610: CVs (or SDs) are great for describing variation in the data; these would be better reported in the results section and interpreted here.

L616: also depends on the severity of the impact, right?

L635-636: do people use smaller mesh size when targeting smaller species? Does this minimize the effect described here?

L666-667: Evaluations and reporting of statistics for these comparisons should be made in the results section.

Figures 10 & 11ab: These should be referenced and described in the results (maybe ~L505).

L692: what is meant by "marginal"?

L698: it is unclear what is meant by “inconsistent weights will cause bias in the analysis”.

L712-713: statements such as “Many clupeids and crustaceans fall under high K category” require citations.

L737: thoughts on the possible mechanism of this relationship between resilience and geographic distribution? Is there evidence of such relationships from other studies?

L761: link to a citation or source or IUCN evaluations.

L766-767: what is meant by “evaluate its usefulness”? How was this done in the manuscript? You showed that it was possible to calculate an index, but I do not see an evaluation of usefulness.

L789-790: advantages/disadvantages of weighted and non-weighted approaches?

L801: seems that interpretation of this metric is thus relative to the fishery being studied. This has implications for comparing IRV to other indices.

L818: not following how 14 species are on the top-10 list.

L833: how often is this list revised, and could your work be used to help India reconsider which species to include on it?

L887: need to describe this “appropriate tool” in a little more detail, so readers can understand how these IRV values for multiple fisheries can be combined.
